# Finite Element Analysis of the Stability of a Sinusoidal Web in Steel and Composite Steel-Concrete Girders

**DOI:** 10.3390/ma13051041

**Published:** 2020-02-26

**Authors:** Krzysztof Śledziewski, Marcin Górecki

**Affiliations:** Faculty of Civil Engineering and Architecture, Lublin University of Technology, Nadbystrzycka 40 Str., 20-618 Lublin, Poland; m.gorecki@pollub.pl

**Keywords:** composite steel–concrete girders, steel girders, sinusoidal web, web buckling, shear stress distribution, finite element analysis

## Abstract

This paper presents the results of numerical investigations into the behavior of a sinusoidal web loaded in shear due to buckling in the period from the onset of buckling until failure, as well as the impact of a reinforced concrete slab on the stability of the web. The analysis concerned steel girders and composite girders with the top flange bonded to a reinforced concrete slab. Nonlinear analyses were performed using the finite element method. The results of the investigations support the conclusion that the appearance and propagation of shear stresses in the sinusoidal web of the composite steel–concrete beam are the same as those in an identical non-composite steel beam, but the bracing of the top flanges improves the shear strength and, at the same time, affects the location of initial stresses. In addition, it was found that, despite the three types of buckling, the predominant failure of the sinusoidal webs, regardless of the presence of the concrete slab, is global buckling. It occurs diagonally through several folds at the same time, including deformation of the web over its entire height.

## 1. Introduction

Conventional plate or box girders contain elements that require a specific approach [1,2]. The central part of the cross section (the web) is usually fairly thin in relation to its height and length due to the need to optimize its weight. This slenderness adversely affects its resistance to buckling. In order to prevent web buckling at fairly small loads, the web must have the required stiffness [3]. One of the ways of doing this is to increase its thickness. It is very common to use transverse and longitudinal stiffeners. There are also ribs in the beams to provide the necessary shear resistance of the beams at critical locations. The simultaneous provision of web stability and shear resistance often leads to an uneven distribution of transverse ribs along the length of the beam. This disturbs the rhythm of the stiffeners, adversely affecting the side appearance of the load-carrying structure, and—most importantly—significantly increases the cost of such girders [4].

The stiffness of the web can also be increased by using profiled corrugated plates, which provide better local stability than girders made of rolled sections and conventional plate girders with a flat web [4,5,6,7,8]. Corrugated plates may have various shapes, e.g., trapezoidal, triangular, channel, cellular, or sinusoidal [9,10,11].

Although beams with profiled webs are widely used in industrial and bridge construction as structural members [12,13,14,15,16], there are still many issues that have to be investigated regarding the configuration of the corrugations [17,18,19,20,21]. One such problem is the mechanisms behind the appearance and propagation of shear stresses and the related change in the strength of sinusoidal webs in steel and composite steel–concrete elements [22,23,24]. In the case of such webs, it is difficult to assess these phenomena due to the significant change in the distribution of strains on a small area of the plate, as well as the fact that the shear strains in corrugated webs are greater than those in flat webs with the same boundary conditions and geometric and material parameters.

The traditional strain gauge measurement methods used in the experimental research allow only local strain measurement. Detailed analysis of the stress distribution over the entire surface of the component requires a large number of measuring points. This does not guarantee that the measuring points indicated will allow for proper analysis. The use of the finite element method, with such a complex geometry as a corrugated web, makes it possible to analyze the stress distribution and its increase over the entire surface under consideration simultaneously and in almost every calculation step.

The purpose of the numerical tests carried out and presented herein in relation to the previous tests carried out by various research teams was primarily to determine the effect of a reinforced concrete slab on the stability of a web with a sinusoidal profile in a steel–concrete composite structure, as well as the form of failure and distribution of shear stresses in such a web. The obtained test results for the steel–concrete composite structure were compared with the results obtained for beams with a sinusoidal corrugated web. Single-span girders loaded in a four-point bend scheme with parameters corresponding to those of the beams subjected to the earlier experimental tests were analyzed [25]. The results of the experimental tests were used as a reference point for the evaluation of numerical test results. All computer simulations were performed using Abaqus 2017 software [26].

## 2. Finite Element Analysis

### 2.1. Properties of the Test Beam

**Figure 1:**
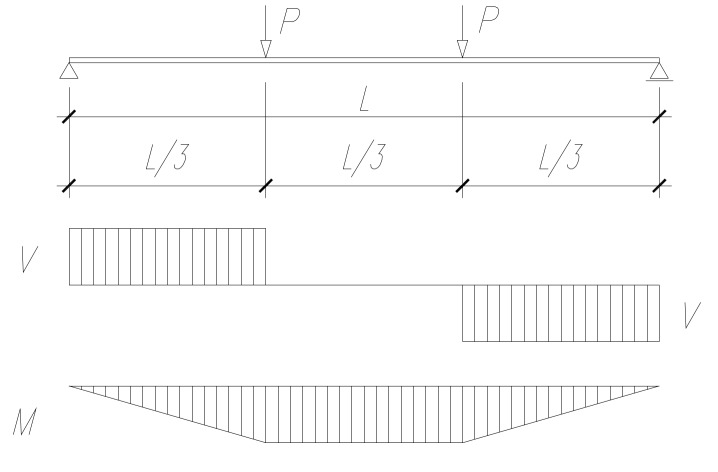
Loading configuration of the analyzed beams—distribution of internal forces.

**Figure 2:**
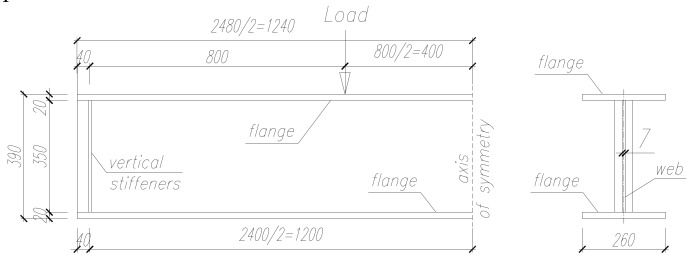
Parameters of the investigated steel beams with a sinusoidal web (dimensions in mm).

**Figure 3:**
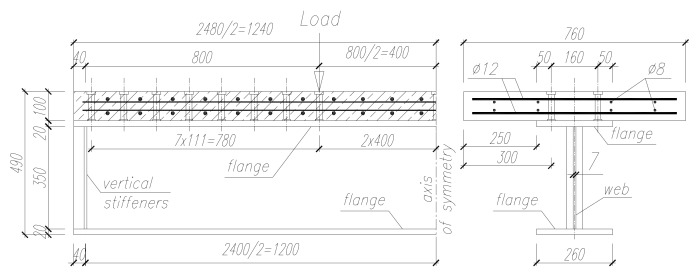
Parameters of the investigated steel beams with a sinusoidal web bonded to a concrete slab (dimensions in mm).

The investigations concerned single-span steel beams in a four-point bending configuration (Figure 1) with a sinusoidal corrugated web (Figure 2), as well as the same configurations with a top flange bonded to a reinforced concrete slab (Figure 3). The results of experimental tests on the analyzed beams were described in [25].

The plate girders had an overall length of 2480 mm, with an effective span of 2400 mm. The top and bottom flange were made of flat plates with a thickness of 20 mm and width of 260 mm. The beam had full-height transverse stiffeners located 40 mm away from its ends, made of flat 10 mm plates, with respective widths of 90 mm and 140 mm.

The height of each plate girder was 390 mm, and the web was 350 mm high and 7 mm thick. The flanges of the girders were joined with the web using double-sided fillet welds with a thickness of 4 mm. The joints between the stiffeners and the web and flanges were also made with double-sided fillet welds with a thickness of 4 mm. The described shape of the beams, including the dimensions of the cross section, prevented local buckling of the flange.

The fact that the compression flange is braced by being bonded to the concrete slab further contributes to the prevention of local buckling. In the composite beams, the plate girder was joined along its entire length with a reinforced concrete slab (with a thickness of 10 cm and width of 76 cm) using two rows of stud fasteners spaced at 160 mm in the cross section. Fasteners with diameter ϕ18 mm and height 100 mm were welded to the top flange of the beam at 111 mm along the axis of the beam, at a section located 800 mm away from each support. Due to the adopted loading configuration to be used in the investigation, the number of fasteners at the 800 mm section in the central area of the beam was limited to two.

The primary reinforcement of the slab consisted of eight ϕ8 mm bars, with four bars arranged along the top and bottom of the slab. Stirrups were made of ϕ12 mm bars.

### 2.2. Geometric Models

The distribution of stresses in the sinusoidal web and impact of the reinforced concrete slab on the stability of the sinusoidal web and failure mode can only be investigated using 3D finite elements [27,28,29,30,31,32]. This can be done using both shell and solid elements. Shell models are highly consistent with experimental measurements, and they do not require modelling of the welds between plate girder components [33,34,35]. All models were constructed in the preprocessor of Abaqus 2017 software [26], which uses the finite element method (FEM).

**Figure 4:**
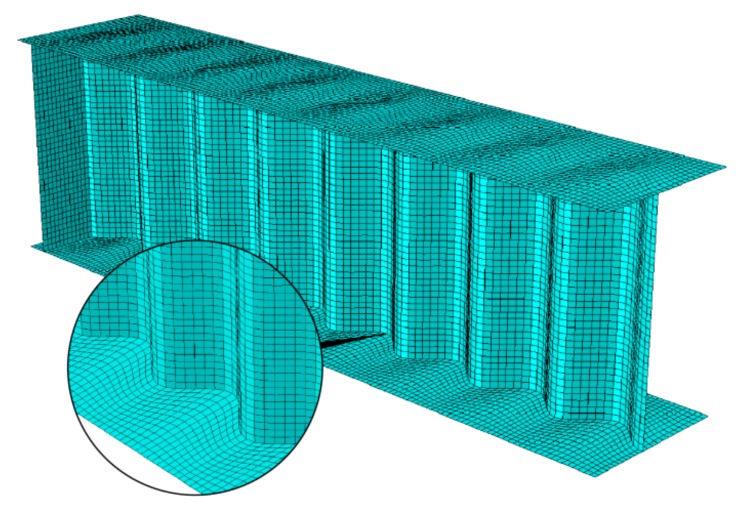
The finite element grid of the I-beam with corrugated web.

**Figure 5:**
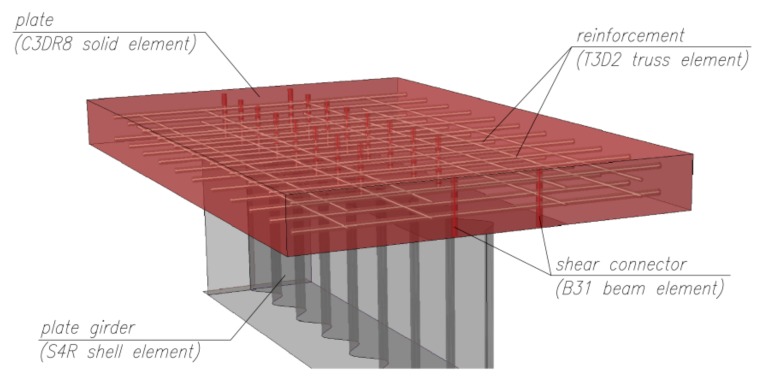
Visualization of the seating of the welding pins and reinforcement in the concrete slab of the beam.

Figure 4 shows the FEM models of the considered beams. A model of half of the beam was created in each of the considered cases. Depending on the analyzed configuration, the models were created using four-node reduced-integration shell elements (S4R—plate girders with stiffeners), eight-node reduced-integration solid elements (C3D8R—concrete slab), two-node linear truss elements (T3D2—primary reinforcement bars and stirrups), and two-node linear beam elements (B31—connecting fasteners). Both the rebars and the rigid fasteners were modelled discretely as elements embedded in the slab attached to the beam, which was the *host* element (Figure 5).

**Figure 6:**
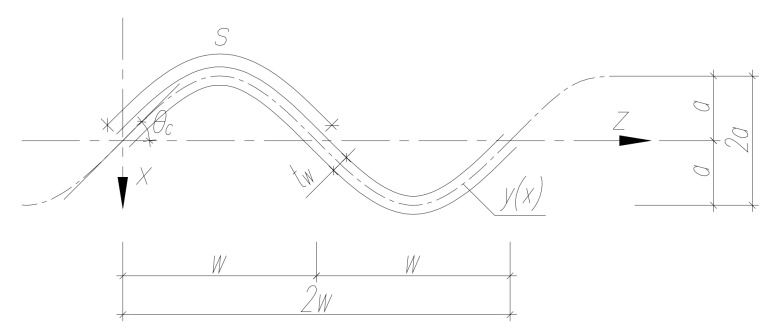
Geometric features of the sinusoidal corrugation.

The corrugation was modelled in accordance with Figure 6, based on Equations (1) and (2) describing the sinusoid curve, with reference to the corrugation amplitude (a = 25 mm) and projected length of half of the corrugation (w = 75 mm):

(1) $$y\left(x\right)=a\;sin\;(\frac{\pi }{w})x$$

(2) $$S\left(x\right)=\int_{0}^{w}\sqrt{1+{\left\{\frac{a\;\pi }{w}\cos\left(\frac{\pi }{w}x\right)\right\}}^{2}}\;dx$$

The contact surfaces between the top flange of the steel beam and the concrete slab were also defined, with coefficient of friction of *µ* = 0.5. The finite element mesh size was approximately 10 mm for each constituent element. In the web, elements with a 1:1 side ratio were used. The beam strips contained elements with side length ratios depending on the location of the elements. In the edge fields, at the edge of the flange, the ratio of lengths of the elements’ sides was about 1:2. In the central part of the flange, the dimensions of the finite elements of the flange were adjusted to the dimensions of the web finite elements. Such a solution was necessary to ensure interaction between the flange shells and the web shell (see Figure 4).

### 2.3. Material Models

**Figure 7:**
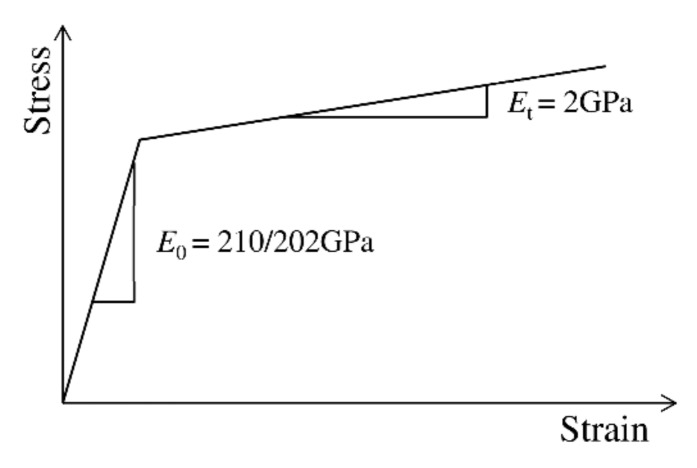
Bilinear steel model.

The elastic–plastic material model was adopted for the constituent elements of the plate girder (Table 1). Based on the yield criterion, the material was subject to the plastic flow rule and kinematic hardening rule (Figure 7). Hardening was determined by adopting a tangent Young’s modulus of *E*_t_ = 0.01*E*.

The concrete, in turn, was described using the Abaqus Concrete Damage Plasticity model (Table 2), which is used for comprehensive modelling of concrete in a complex stress state [36,37,38,39].

This model includes combinations of nonassociated plasticity with hardening and scalar isotropic elastic damage to determine irreversible changes that occurred during the loading and unloading cycles. The CDP model is based on the brittle–plastic degradation model created by Lubliner et al. [40,41] and later perfected by Lee and Fenves [42], which assumes that the two main mechanisms of destruction are cracking as a result of stretching and crushing of the concrete under compression.

**Figure 8:**
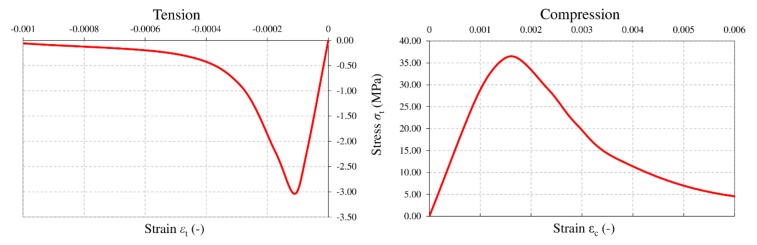
Physical laws of the concrete modelled in the numerical calculations.

In the FEM analysis, the Young’s modulus of concrete was adopted as *E*_0_ = 32 GPa and the Poisson ratio as *v* = 0.17. Figure 8 shows the parameters defining the nonlinear behavior of concrete, in both the tension and compression zones. The physical laws of concrete were described using the Saenz function [43].

### 2.4. Loading Method and Boundary Conditions

**Figure 9:**
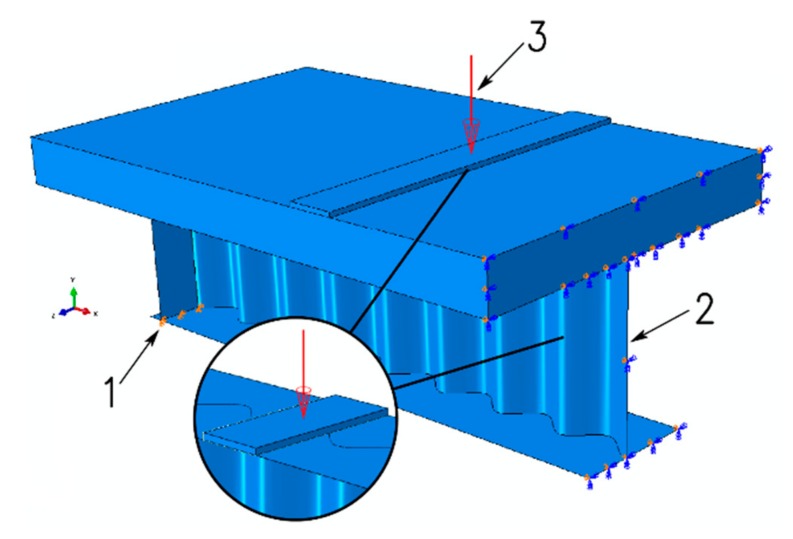
Boundary conditions adopted in the calculations for the considered conditions.

The loading points for external loads were selected as for the standard four-point beam bending configuration, in such a way that the bending moment was constant along the 800 mm section in the middle of the beam (see Figure 1). In the numerical calculations, the concentrated load (labelled “3” in Figure 9) was applied perpendicularly to the loaded surface through an intermediate perfectly rigid body.

The beam was supported linearly, precisely at the stiffeners welded to the profile, which prevented local yielding upon exposure to highly concentrated reaction forces. Vertical displacements in the *y* direction and horizontal displacements in the *z* direction were locked at the location of the support (labelled “1”). Horizontal displacement in the *x* direction and rotation about the *z* and *y* axes, in turn, were locked at point “2”.

### 2.5. Validation of Numerical Models

All calculations considered both the physical nonlinearity describing the applied materials and the geometric nonlinearity resulting from high deformation. The numerical models were verified based on the list of calculated displacements, ultimate loads, and failure modes of the investigated beams and numerical model.

**Figure 10:**
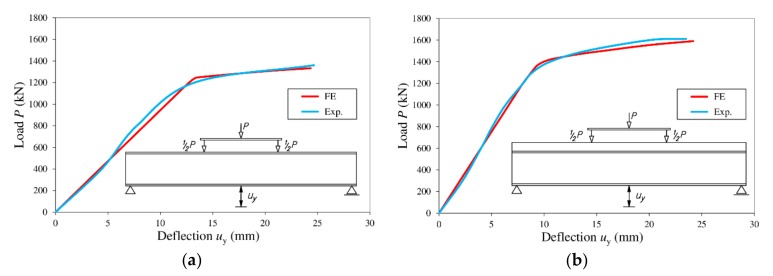
Comparison of vertical displacements determined through experimental tests and numerical analysis: (**a**) steel beam; (**b**) steel beam bonded to a reinforced concrete slab.

The deflection was read in the vertical plane of the cross section, in the middle of the span on the external surface of the lower flange (*u*_y_). The highest displacements determined in the numerical analysis (*u*_ulty,FE_) are consistent with the results of laboratory tests (*u*_ulty,Exp_). The largest difference between the calculated and tested deflections (Figure 10) was 3%.

**Figure 11:**
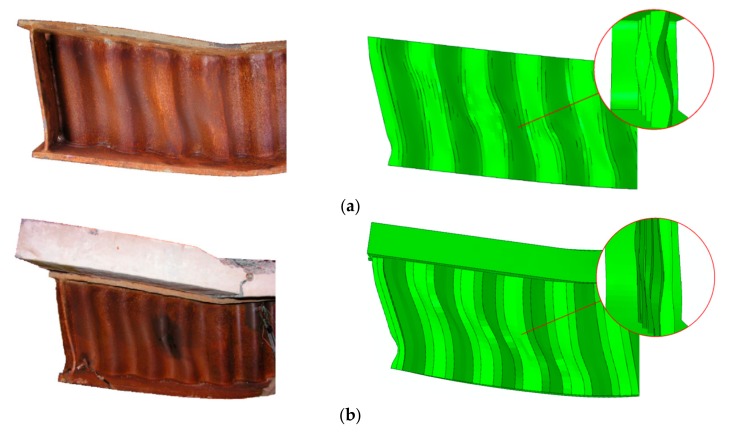
Comparison of failure modes caused by global buckling of the web determined through experimental tests and numerical analysis: (**a**) steel beam; (**b**) beam bonded to a reinforced concrete slab.

The consistency of the experimental and numerical results is also noticeable in the ultimate external loads (*P*ult) and ultimate failure modes of the beams (Figure 11). The ratio of the ultimate load determined through numerical analysis (*P*_ult,FE_) to the experimental ultimate load (*P*_ult,Exp_) was 0.98 for the steel beam and 0.99 for the steel beam bonded to a reinforced concrete slab. In both cases, failure occurred in the area of interaction of the bending moment and shear force, due to buckling causing the appearance of a yield line in the web.

The failure mode clearly demonstrates global buckling of the web. Buckling in the individual corrugations noticeably disappears towards the end of the beam. The same phenomenon was observed during experimental tests [25].

The specific ultimate loads determined through experimental tests and numerical investigations are given in Table 3.

Comparative analyses indicate good consistency of the results of experimental tests and numerical analysis, as well as a general consistency of the computational models with the assumptions concerning material strength theories. The representative numerical models prepared based on the results of experimental tests were used for further parametric investigations.

## 3. Analysis Results and Discussion

Simulations of the post-buckling behavior of the structure (in post-critical states) were carried out using the RIKS analysis available in the Abaqus software [26], which enables “passing” through the bifurcation point. This method finds the point of equilibrium at the end of each step. In this way, it is possible to not only increase (as in the conventional iterative method) but also decrease the force in order to meet the static equilibrium criterion. The size of the calculation step in the modified RIKS method [44] depends on the “Arc Length”, measured along the static equilibrium path in the load–displacement space.

Finite element analysis indicated that the appearance and propagation of shear stresses in the sinusoidal web are similar in both of the considered configurations, with the difference lying in the location of the point where initial stresses are induced.

**Figure 12:**
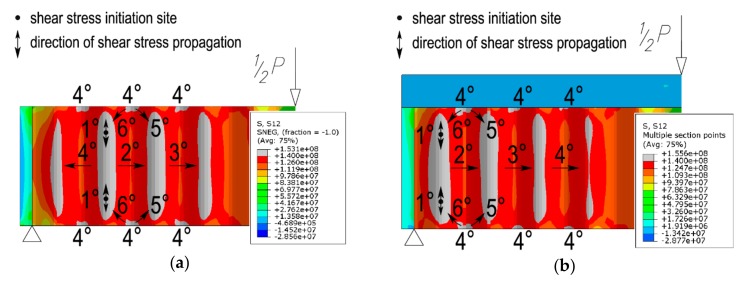
Mode of propagation of shear stresses with increasing load in the sinusoidal web (unit: Pa): (**a**) steel beam; (**b**) beam bonded to a reinforced concrete slab.

As shown in Figure 12, stresses were initiated near the end of the beam (which corresponds to the final failure mode, see Figure 11) simultaneously in the top and bottom areas of the beam, at ¼ of the web height (from the bottom and top flange). With increasing load, the stresses propagate accordingly towards the neutral axis of the beam cross section and towards the flanges (direction 1°) and also to adjacent corrugations (directions 2° and 3°). The last stage in the appearance of noticeable stresses is the place located at the edge of the web, at the top of the corrugation near the interface with the flanges (direction 4°), and further propagation towards the stress areas formed earlier (directions 5° and 6°).

In the steel beam bonded to the reinforced concrete slab, the initial shear stresses appear in and propagate from the first corrugation located closest to the support stiffener (Figure 12b). In the non-composite steel beam, the place of initiation of shear stresses is located differently than in the composite beam because the stresses originate in more distant corrugations, located slightly further away from the support stiffener (Figure 12a).

**Figure 13:**
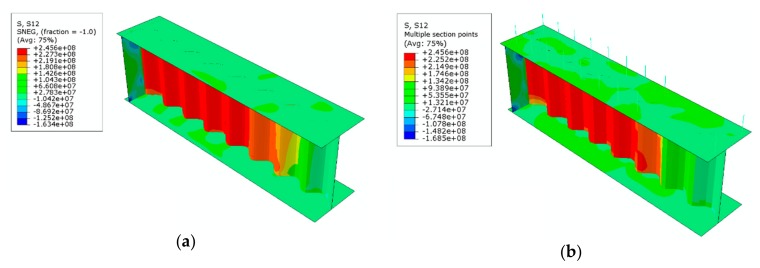
Pre-buckling shear stress distribution in the sinusoidal web (unit: Pa): (**a**) steel beam; (**b**) beam bonded to a reinforced concrete slab (view without the slab).

The analyses also confirmed that in the configuration with generation of the bending moment, where normal compressive or tensile stresses appear in the extreme fibers of the cross section (regardless of the type of cross section), the negligible longitudinal stiffness of the sinusoidal web does not have an impact on the mutual strain change in the top and bottom flanges. The value of shear stresses in the flanges is approximately zero, which is why the entire shear load is transferred only through the web (Figure 13). This is confirmed by the occurrence of the so-called accordion effect in I-beams with a corrugated web [45].

**Figure 14:**
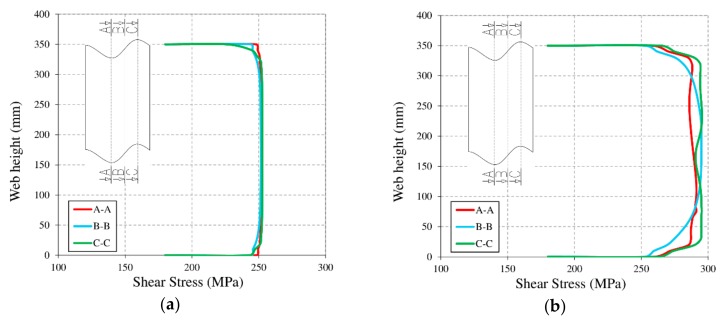
Typical σ_12_ shear stress distribution on the corrugated web at three different corrugation cross sections: (**a**) before buckling; (**b**) after buckling.

During the numerical investigations, it was also observed that the shear stress distribution at the corrugated web was the same in all analyzed beams (Figure 14). Shear stresses were distributed evenly onto the corrugated web before buckling, and they reached the highest level just after the appearance of buckling [11]. The post-buckling stress distribution itself varies depending on the location of the cross section on the corrugation.

**Figure 15:**
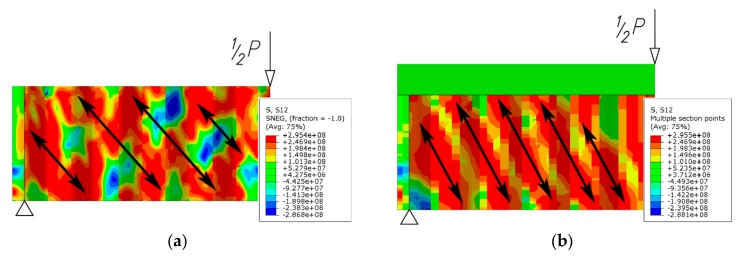
Final post-buckling shear stress distribution in the sinusoidal web (unit: Pa): (**a**) steel beam; (**b**) beam bonded to a reinforced concrete slab.

In the A–A and C–C sections (locations: amplitude of the corrugation), the shear stresses reached an almost constant level within a range of approximately 80% of the web height. In the B–B section (location: at the axis between adjacent amplitudes), they only include an area of approximately 45%, and they gradually decrease towards the flanges, eventually reaching pre-buckling levels. This is directly related to the appearance of yield lines (see Figure 15).

The maximum levels of shear stresses in the web after buckling were approximately 295 MPa. Although the values are comparable for each of the considered configurations, they were reached at different stress ratios. This is related to the presence of bracing in the form of the reinforced concrete slab bonded to the top flange of the plate girder.

The shear stress distribution of the models indicates that the highest shear stresses are centered along diagonal lines over the entire height of the web, which suggests the appearance of yield lines due to global buckling. It should also be noted that the yield lines do not propagate to the top flanges of the plate girder. This behavior is different from what can be observed in conventional solutions with a flat web [11], where yield lines appear near the applied load, immediately next to the interface of the flange with the web.

**Figure 16:**
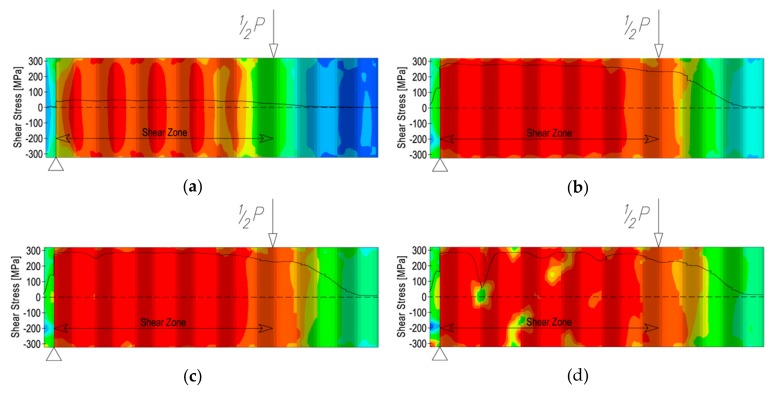
Shear stress distribution in the sinusoidal web of the steel beam with rising load: (**a**) at the beginning of loading; (**b**) before buckling; (**c**) upon formation of the first plastic hinge; (**d**) after buckling.

**Figure 17:**
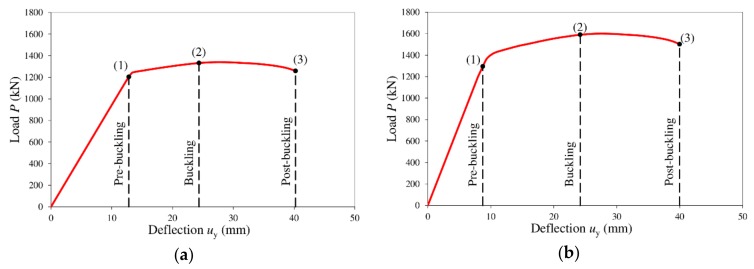
Static equilibrium paths determined in the numerical investigations: (**a**) steel beam; (**b**) beam bonded to a reinforced concrete slab.

A change in the stiffness of the web in the transverse direction and appearance of yield lines also affects the shear stress distribution along the sinusoidal web (Figure 16) with rising load (Figure 17).

During the first stage of loading before buckling, shear stresses are distributed uniformly along the web (Figure 16a). With increasing load, shear stresses rise linearly (Figure 16b) until the formation of the first plastic hinge (Figure 16c). At this point, the shear stresses reach the maximum level. A continued load increase results in the formation of further places where buckling appears. At the buckling points, the shear stresses quickly decrease (Figure 16d). After buckling, the shear stresses locally disappear. This process occurs from the direction of the supports towards the axis of the beam.

The propagation of shear stresses is the same for the steel beam and for the steel beam bonded to the reinforced concrete slab. It was also observed that in both cases, the extent of the area with shear stresses, equal to the height of the web, included ¾ of the area of the constant bending moment. This is the opposite of what happens in statically determinate beams in a four-point bending configuration, where shear stresses rapidly disappear just outside of the plane determined by the place of application of the loading force (see Figure 1).

The analyses support the previous observations of various authors, which indicated that shear displacements in a corrugated web are the cause of nonlinearity and an increase in the global displacements of the entire structure [10,12,46,47], and shear buckling of the corrugated web results in the appearance of yield lines [48]. Figure 17 shows the load–vertical displacement relationships determined during the investigations at the midspan of each analyzed beam.

Characteristic points (1), (2), and (3) in Figure 17 separate the following areas: area 0–1 with reversible displacements of both the web and the flanges (elastic range), area 1–2 with the formation and increase of tension fields and yield lines in the web, and area 2–3 leading to the buckling and failure of the web.

Area 2–3 is the place where the impact of elastic–plastic displacements from bending accumulates. The rising accumulation of displacements from bending occurs along with the increase of loads and propagation of tension fields (Figure 15). With the appearance of yield lines, the impact of shear strains of the web on the nonlinear global static equilibrium path becomes significant. The appearance of yield lines clearly separates the quasilinear part of the displacements from nonlinear displacements (point 1 in Figure 17a,b).

An increase of the load above the critical shear strength results in global web buckling. After the maximum load is reached and point (2) is exceeded, vertical displacements *u*_y_ increase, with a simultaneous decrease of load levels. At this stage, yield lines are also formed in the web. At point (3), in both cases, the yield lines propagate, leading to the failure of the girder.

**Figure 18:**
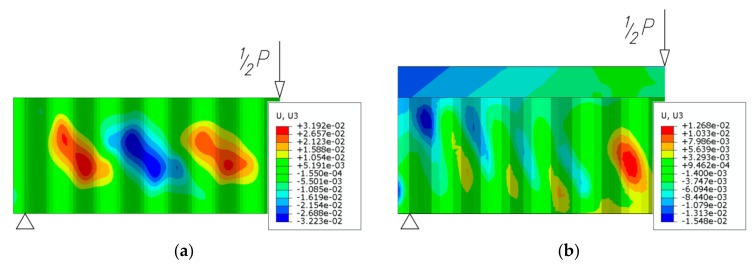
Final distribution of displacements *u*_z_ in the transverse plane of the web (unit: m): (**a**) steel beam; (**b**) beam bonded to the reinforced concrete slab.

Final failure occurs in the extreme zones of the beam, i.e., in the fields where the shear force reaches the highest level and the bending moment disappears towards the support. The highest deformations can be observed in the cross section of the beam nearest to the point of application of the loading force (Figure 18). This is indicative of failure due to the presence of high bending moments with the simultaneous presence of shear force.

## 4. Conclusions

Previous studies focused mainly on estimation methods and the impact of various geometric parameters of the web on its load-bearing capacity as a result of shear forces. The purpose of the presented research was to determine the impact of a reinforced concrete slab on sinusoidal web stability in steel–concrete composite elements, as well as to determine the form of destruction of such structures together with the method of shear stress generation and propagation. The obtained test results were compared with those of identical steel beams with a sinusoidal web but without the concrete slab. Due to the lack of possibility to examine the global picture of shear stress distribution in the entire area of their occurrence and in the time interval during experimental research, geometric and material nonlinear finite elements analysis was performed.

As a result of the analysis, we found the following:

- The dominant failure of sinusoidal webs, regardless of the presence of the concrete slab, was global buckling, which extended diagonally across several corrugations at the same time, including deformation of the web over its entire height; that is the reason why, in accordance with the hypothesis of M. T. Huber, such webs can be classified as orthotropic plates where orthotropy is caused not by the material but by the geometric properties of the configuration;
- A change in the location of the neutral axis of the cross section in the composite steel–concrete beam relative to the steel beam has an impact on the location of initial shear stresses; in the case of the composite beam, shear stresses are propagated from the cross section located closest to the support, and in the noncomposite beam, with a uniform steel cross section, the center of the formation of shear stresses is located in the central area of the field with a constant level of internal shear forces. Further propagation of the stresses is the same in the case of the nonbonded steel beam with a sinusoidal web and in the case of the beam bonded to a concrete slab;
- The shear strength of the composite beam relative to the steel beam when the parameters of the steel cross section are the same is approximately 15% greater; the conventional method of determining the shear strength in composite steel–concrete beams, in accordance with which shear strength depends on the steel web, seems too conservative for the assessment of composite steel–concrete beams with a corrugated web;
- Deflections of the composite steel–concrete beam in the elastic range are lower than those in the steel beam due to the stiffness of the entire configuration, and a quick rise in deflections can be observed in the range where yield lines appear in the corrugated web.

## Figures and Tables

**Table 1:** Material properties of steel in the numerical model.

Material Characteristics	Flanges	Stiffeners	Web	Connecting Fasteners	Rebars
Yield point (Mpa)	501.0	501.0	434.0	350.0	410.0
Tensile strength (Mpa)	591.0	591.0	512.0	450.0	550.0
Young’s modulus (Gpa)	210.0	210.0	202.0	210.0	210.0
Shear modulus (Gpa)	81.0	81.0	78.0	81.0	81.0
Poisson ratio (–)	0.3	0.3	0.3	0.3	0.3

**Table 2:** Parameters of the Concrete Damage Plasticity model.

Internal Friction Angle *β*	Eccentricity of the Plastic Potential *є*	*f*_b0_/*f*_c0_	Shape of the Plastic Potential Surface *K*_c_	Viscosity Parameter *μ*
36°	0.1	1.16	0.667	0

**Table 3:** Comparison of the determined ultimate values: *P*_ult_ and *u*_ult_.

Object under Test	*P*_ult_ (kN)		*u*_ult_ (mm)	
	Exp.	FE	Exp.	FE
Steel beam	1360.49	1332.71	24.65	24.35
Composite steel–concrete beam	1611.28	1590.80	23.52	24.19
